# Linked color imaging improves polyp miss rates in total colonoscopy in a multicenter randomized back to back trial

**DOI:** 10.1038/s41598-025-20633-2

**Published:** 2025-10-21

**Authors:** Ryo Shimoda, Daisuke Yamaguchi, Kazutoshi Hashiguchi, Kazuhiro Mizukami, Akira Aso, Takashi Akutagawa, Koichi Miyahara, Tetsuro Honda, Keiichi Hashiguchi, Tetsuya Ohira, Kensuke Fukuda, Masayuki Kabayama, Hideaki Miyamoto, Ryosuke Gushima, Yorinobu Sumida, Sho Suzuki, Fumisato Sasaki, Naoyuki Yamaguchi, Tetsu Kinjo, Tadashi Miike, Ken Ohnita, Tomohiko Moriyama, Shin Fujioka, Takashi Shono, Shimpei Shirai, Kensei Ohtsu, Fumiaki Kiyomi

**Affiliations:** 1 Department of Endoscopic Diagnostics and Therapeutics, Saga University Hospital, 5-1-1 Nabeshima, Saga, 849-8501 Japan; 2 Division of Gastroenterology, Department of Internal Medicine, Faculty of Medicine, Saga University, Saga, Japan; 3 Department of Gastroenterology, National Hospital Organization Ureshino Medical Center, Ureshino, Japan; 4 Interventional Endoscopy Center, Josuikai Imamura Hospital, Tosu, Japan; 5 Department of Gastroenterology, Oita University, Yufu, Japan; 6 Department of Gastroenterology, Aso Clinic, Fukuoka, Japan; 7 Department of Internal Medicine, Karatsu Red Cross Hospital, Karatsu, Japan; 8 Department of Gastroenterology, Nagasaki Harbor Medical Center, Nagasaki, Japan; 9 Department of Endoscopy, Nagasaki University Hospital, Nagasaki, Japan; 10 Department of Endoscopy, Ryukyu University Hospital, Nakagami, Japan; 11 Department of Digestive and Lifestyle Diseases, Graduate School of Medical and Dental Sciences, Kagoshima University, Kagoshima, Japan; 12 Department of Gastroenterology and Hepatology, Kumamoto University Hospital, Kumamoto, Japan; 13 Department of Gastroenterology, National Hospital Organization Kyushu Medical Center, Fukuoka, Japan; 14 Department of Gastroenterology and Hepatology, Division of Endoscopy, Center for Digestive Disease, University of Miyazaki Hospital, Miyazaki, Japan; 15 Department of Gastroenterology and Hepatology, Shunkaikai Inoue Hospital, Nagasaki, Japan; 16 International Medical Department, Kyushu University, Fukuoka, Japan; 17 Department of Medicine and Clinical Science, Graduate School of Medical Sciences, Kyushu University, Fukuoka, Japan; 18 Department of Gastroenterology, Kumamoto Chuo Hospital, Kumamoto, Japan; 19 Department of Internal Medicine, Karatsu Saiseikai Hospital, Karatsu, Japan; 20 Department of Gastroenterology, Fukuoka University, Chikushi Hospital, Fukuoka, Japan; 21 Department of Statistics and Data Center, Clinical Research Support Center Kyushu, Fukuoka, Japan

**Keywords:** LCI, Colonoscopy, Polyps, ADR, Miss rate, Gastroenterology, Risk factors

## Abstract

Linked color imaging (LCI) was developed to detect gastrointestinal neoplasms. The current study aimed to determine whether the use of LCI, compared with white-light imaging (WLI), can improve the miss rates of colorectal polyp. A multicenter, randomized back-to-back study was conducted in 16 Japanese endoscopy units. Patients were randomized according to examination: tandem colonoscopy with WLI followed by LCI (WLI-LCI group) and with LCI followed by WLI (LCI-WLI group). The detected polyps were evaluated according to location, size, morphology, and histopathological diagnosis. The primary outcome was polyp miss rate per patient (PMR-PP) in total colonoscopy. The secondary outcome was adenoma detection rate (ADR) during the first assessment in each group. The full analysis set comprised 327 participants, and 320 were included in either two groups. The PMR-PPs were 9.3% and 20.6% in the LCI-WLI and WLI-LCI groups, respectively. Regarding location, the PMR-PP of LCI was significantly lower than that of WLI in the transverse and descending colons and rectum. In terms of diminutive adenomas (< 5 mm), the ADR of LCI (38.2%) was significantly higher than that of WLI (29.1%). LCI was superior to WLI in terms of polyp miss rate particularly in the transverse and descending colons and rectum.

**Supplementary Information:**

The online version contains supplementary material available at 10.1038/s41598-025-20633-2.

## Introduction

Currently, colonoscopy is the gold standard for detecting and treating colorectal cancer (CRC) and is effective in reducing the incidence and mortality rates of this condition. The detection and resection of colorectal adenomas via endoscopy can reduce the risk of CRC^1,2^. However, previous studies have shown that the adenoma miss rates of white-light imaging (WLI) range from 10% to 30%^3–5^.

To improve the detection of colorectal adenoma via colonoscopy, various devices and methods have been used. These include the assessment of areas behind colonic folds, increased inspection time, pan-colonic dye spraying^6^, cap-assisted colonoscopy^7,8^ and use of a newly developed colonoscope (e.g., wide-angle colonoscopy^9,10^, Third Eye colonoscopy^11,12^, and full-spectrum endoscopy [FUSE])^13,14^.

In the last 10 years, the detectability of CRC using several IEE), such as narrow-band imaging (NBI)^15–17^, blue light imaging (BLI)^18–20^, flexible spectral imaging color enhancement (FICE)^21,22^, and auto fluorescence imaging (AFI)^23^, was evaluated. However, Chung et al.^21^ and Aminalai et al.^22^ reported that FICE, compared with WLI, has no advantages in terms of improving adenoma miss rate (AMR) and adenoma detection rate (ADR). A recent meta-analysis of completed study did not show the superiority of NBI over the standard WLI^24^. Horimatsu et al.^16^ revealed that second-generation NBI, compared with WLI, is advantageous in terms of the mean number of detected polyps per patient. Ikematsu et al.^19^ showed that the mean number of detected adenomas per patient (MAP) in BLI was higher than that in WLI. Recently, Takeuchi et al. revealed that AFI was effective in detecting flat neoplasms in the right side of the colon^23^. Based on the results of previous reports, the efficacy of IEE is controversial, and whether IEE can improve the detection of colorectal neoplasms remains unclear.

**Fig. 1:**
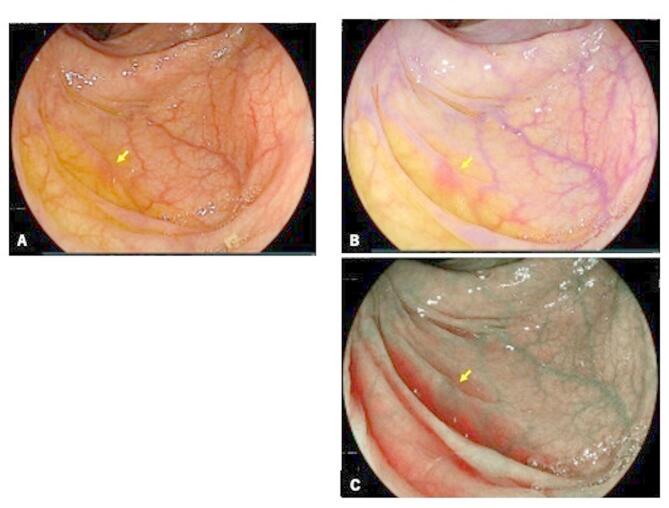
Representative endoscopic findings of diminutive adenoma. (**a**–**c**) show a case of diminutive adenoma on WLI, LCI, and BLI, respectively (with arrow).

Linked color imaging (LCI) is newly developed IEE that can effectively detect gastrointestinal neoplasms by contrast enhancement between neoplasms and the surrounding normal mucosa (Fig. 1). The technology comprises pre-procession by two narrow-band lasers and post-procession by optical enhancement. Digital image post-procession emphasizes slight color differences and provides a better contrast within the red color range. Although the reddish and whitish mucosa are emphasized, natural tones do not change significantly. The expansion of color contrast between gastrointestinal lesions and the surrounding mucosa improves visibility. Several studies have reported the efficacy of LCI in assessing the visibility^25^ and detectability^20,26–35^ of colorectal tumors. However, the efficacy of LCI in detecting colorectal polyps in the whole colon after stratifying based on lesion location, size, and morphology was not evaluated in a multicenter, prospective study.

Thus, we conducted a multicenter, randomized back-to-back study to determine whether LCI, compared with WLI, can improve polyp miss rate per patient (PMR-PP) in each colon segment.

## Results

### Characteristics of patients

**Fig. 2:**
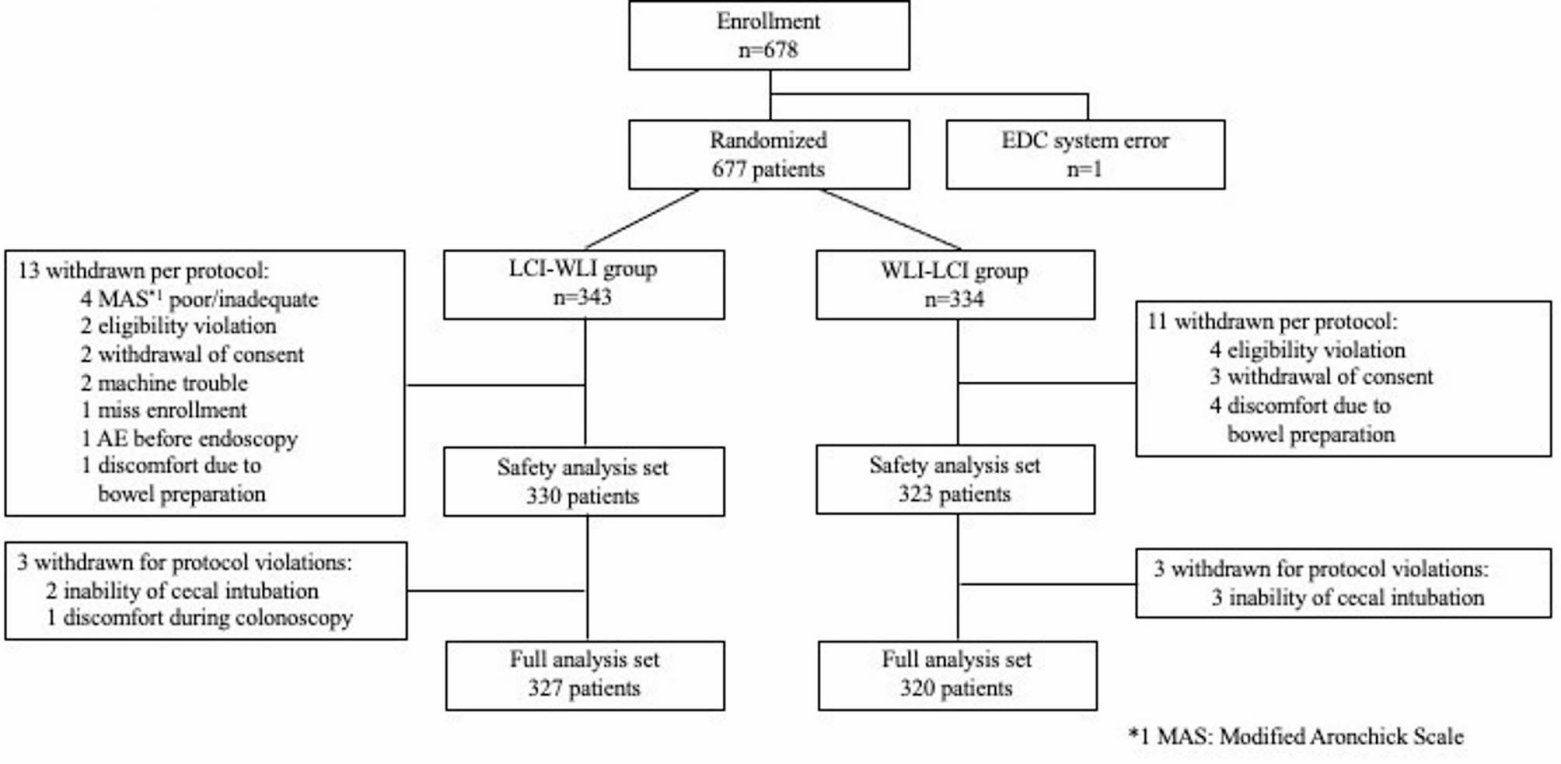
Flow of participant enrollment *1 MAS: Modified Aronchick Bowel Preparation Scale *2 AE: Adverse event.

Among 678 Patients enrolled, 677 patients were randomly classified under the LCI-WLI group (*n* = 343) or the WLI-LCI group (*n* = 334) from November 2018 to May 2020. The enrollment was completed after including 400 patients with colorectal polyp. After randomization, 24 patients withdrew before colonoscopy due to various reasons (*n* = 6, eligibility violation; *n* = 5, withdraw of consent; *n* = 5, discomfort due to bowel preparation; *n* = 4, poor bowel preparation; and *n* = 5, others). Furthermore, discontinuation of examination occurred in three patients in each group. The full analysis sets were 327 and 320, and patients without polyp information who dropped out from the study were excluded from the safety analysis set (Fig. 2). The two groups did not differ in terms of sex, age, family history of CRC, indication for examination, sedation, and Modified Aronchick Scale^36^ and Boston Bowel Preparation Scale (BBPS) scores^37^ (Table 1). Table S1 shows Modified Aronchick Scale. This scale evaluates the entire intestinal tract reported in descending order of cleansing degree on a 5-point scale: excellent, good, fair, poor, and inadequate.

**Table 1:** Baseline characteristics of the participants.

	LCI-WLI group	WLI-LCI group	*P*-value
	*n* = 327	*n* = 320	
Male sex	186 (56.9)	187 (58.4)	0.689
Age (years), SD	61.2 (12.1)	61.9 (11.2)	0.441
Family history of CRC	28 (8.6)	33 (10.3)	0.454
Indication for examination			
FIT- positive	75 (22.9)	67 (20.9)	0.973
Detailed examination	215 (65.7)	218 (68.1)	
Polyp surveillance	8 (2.4)	7 (2.2)	
Any abdominal symptoms	18 (5.5)	18 (5.6)	
Others	11 (3.4)	10 (3.1)	
Sedation	252 (77.1)	237 (74.1)	0.374
Modified aronchick scale			
Excellent	174 (53.2)	188 (58.8)	0.178
Good	115 (35.2)	108 (33.8)	
Fair	38 (11.6)	24 (7.5)	
Poor/inadequate	0 (0.0)	0 (0.0)	
BBPS, median (min–max)	8 (2–9)	9 (0–9)	0.288

Data were presented as number of patients (%), unless specified otherwise.

SD: standard deviation; CRC: colorectal cancer; BBPS: Boston Bowel Preparation Scale.

*P*-value: Chi-square test for nominal scale variables, Wilcoxon rank sum test for ordered scale/non-normality variables, and student’s *t*-test for continuous variables.

### Details of the polyp

In total, 1177 polyps were detected in the LCI-WLI (620 lesions) and WLI-LCI (557 lesions) groups. The clinical features of the lesions (location, size, morphology, and histopathologic findings) are shown in Table 2. The total number of polyps detected during the first observation was 327 in the LCI-WLI group and 320 in the WLI-LCI group (Table S2). Most polyps were removed endoscopically: 57/64 biopsies, 371/348 hot/cold snare polypectomy, 98/107 endscopic mucosal resection (EMR) and 14/12 endoscopic submucosal resection (ESD). However, 81 lesions in the LCI-WLI group and 28 in the WLI-LCI group were not diagnosed pathologically because they were missed during treatment or the resected lesions were not retrieved.Lesions that the endoscopist clearly diagnosed as hyperplastic polyps were not counted and were not resected.

**Table 2:** Number of detected polyps (sum of first and second observations).

	LCI-WLI group	WLI-LCI group	*P*-value
Total number of polyps	620	557	
Location			
Ascending colon	183 (29.5)	163 (29.3)	0.602
Transverse colon	161 (26.0)	140 (25.1)	
Descending colon	68 (11.0)	67 (12.0)	
Sigmoidal colon	165 (26.6)	136 (24.4)	
Rectum	43 ( 6.9)	51 ( 9.2)	
Size			
< 5 mm	342 (55.2)	302 (54.2)	0.368
5–10 mm	216 (34.8)	185 (33.2)	
> 10 mm	62 (10.0)	70 (12.6)	
Morphology			
0-Is	426 (68.7)	366 (65.7)	0.027
0-Isp	52 (8.4)	69 (12.4)	
0-Ip	14 (2.3)	24 (4.3)	
0-IIa	127 (20.5)	98 (17.6)	
0-IIb	0 (0.0)	0 (0.0)	
0-IIc	1 (0.2)	0 (0.0)	
Histopathology			
Adenoma with low-grade dysplasia	505 (81.5)	474 (85.1)	< 0.001
Adenoma with high-grade dysplasia	7 (1.1)	11 (2.0)	
Invasive cancer	1 (0.2)	4 (0.7)	
TSA	4 (0.6)	10 (1.8)	
SSA/P	22 (3.5)	30 (5.4)	
Hyperplastic polyp	0 (0.0)	0 (0.0)	
ND	81 (13.1)	28 (5.0)	

Data were presented as number of polyps (%).

ND: pathologic examination was not performed.

*P*-value: Chi-square test or Fisher’s exact test for nominal scale variables and Wilcoxon rank sum test for ordered categorical variables.

### Withdrawal time

The withdrawal time in LCI was calculated by summing up the first observation in the LCI-WLI group and the second observation in the WLI-LCI group in all segments of the colon. That in WLI was calculated by summing up the second observation in the LCI-WLI group and the first observation in the WLI-LCI group in all segments of the colon. The withdrawal time in LCI was significantly longer than that in WLI (6.66 vs. 6.38 min, *P* = 0.016) (Table 3).

**Table 3:** Withdrawal time of each procedure (LCI/WLI).

	LCI (*n* = 327) LS mean	WLI (*n* = 320) LS mean	Difference in LCI-WLI (95% CI)	*P*-value
Ascending colon	1.37	1.37	0.00 (-0.09–0.10)	0.960
Transverse colon	1.65	1.53	0.12 (0.03–0.21)	0.009
Descending colon	1.06	1.07	-0.01 (-0.06–0.05)	0.807
Sigmoidal colon-rectum	2.58	2.42	0.15 (0.04–0.27)	0.009
Total	6.66	6.38	0.27 (0.05–0.49)	0.016

LS mean: least square mean. The unit is minutes.

P-value: Cross-over ANOVA (LCI-WLI/WLI-LCI), observation (first/second observation), and imaging (LCI/WLI) as fixed effects and subject as a random effect.

### Polyp miss rates per patient

The PMR-PPs were 9.3% and 20.6% in the LCI-WLI and WLI-LCI groups, respectively. Hence, the results significantly differed (Wilcoxon test, *P* < 0.001) (Table 4). Furthermore, results were calculated by stratifying according to location, size, morphology, and histopathological findings. Regarding location, the PMR-PPs of the LCI-WLI group were significantly lower than that of the WLI-LCI group in the transverse and descending colons and rectum, respectively (3.9% vs. 11.1%, *P* < 0.001; 2.7% vs. 7.3%, *P* = 0.008; and 0.9% vs. 3.1%, *P* = 0.028). In the ascending and sigmoid colons, the two groups did not differ (4.6% vs. 8.3%, *P* = 0.066; 4.7% vs. 6.7%, *P* = 0.388). In small lesions (< 5, 5–10 mm), the PMR-PPs of the LCI-WLI group were significantly lower than those of the WLI-LCI groups (9.3% vs. 17.0%, *P* = 0.001; 4.3% vs. 10.6%, *P* = 0.003). In large lesions (> 10 mm), there were no difference in the PMR-PPs between the LCI-WLI and WLI-LCI groups (0.2% vs. 1.1%, *P* = 0.095). In terms of morphology, 0-Is and 0-IIa lesions were missed less often in the LCI-WLI group than in the WLI-LCI group (7.2% vs. 17.5%, *P* < 0.001; 2.5% vs. 6.0%, *P* = 0.024). Regarding histopathological findings, the PMR-PPs not only of conventional adenoma but also of SSA/P were significantly lower in the LCI-WLI group than in the WLI-LCI group (conventional adenoma: 9.8% vs. 20.9%, *P* = 0.001; SSAP: 0.3% vs. 1.6%, *P* = 0.031). Thirty-nine experts conducted 622 examinations (LCI-WLI group: *n* = 313, WLI-LCI group: *n* = 309), while 6 non-experts conducted 25 examination (LCI-WLI group: *n* = 14, WLI-LCI group: *n* = 11). Table S3 shows the PMR-PP in expers. In expert, the PMR-PPs were 9.6% and 21.0% in the LCI-WLI and WLI-LCI groups, respectively. Hence, the results significantly differed (Wilcoxon test, *P* < 0.001). Table S4 shows the PMR-PP in non-experts. In non-experts, no difference in PMR-PP was observed between the LCI-WLI group and the WLI-LCI group (3.6% vs. 7.6%, *P* = 0.438). Regarding PMR-PP of non-expert, no significant differences were observed even when analyzing by location, morphology, or histopathology of polyp. Furthermore, the polyps discovered by non-specialists did not include type 0-II or SSA/P flat polyps.

**Table 4:** Polyp miss rates (%) per patient (SD).

	LCI-WLI group	WLI-LCI group	*P*-value
	*n* = 327	*n* = 320	
All polyps	9.3 (23.6)	20.6 (34.0)	< 0.001
Location			
Ascending colon	4.6 (19.0)	8.3 (26.4)	0.066
Transverse colon	3.9 (17.8)	11.1 (29.4)	< 0.001
Descending colon	2.7 (15.2)	7.3 (25.3)	0.008
Sigmoidal colon	4.7 (19.4)	6.7 (23.5)	0.388
Rectum	0.9 (9.5)	3.1 (17.0)	0.028
Size			
< 5 mm	9.3 (25.8)	17.0 (33.8)	0.001
5–10 mm	4.3 (17.7)	10.6 (28.1)	0.003
> 10 mm	0.2 (3.7)	1.1 (9.9)	0.095
Morphology			
0-Is	7.2 (20.6)	17.5 (33.6)	< 0.001
0-Isp	0.9 (9.5)	2.0 (12.7)	0.077
0-Ip	0.0 (0.0)	0.3 (5.6)	0.312
0-IIa	2.5 (13.8)	6.0 (22.1)	0.024
0-IIb	0.0 (0.0)	0.0 (0.0)	-
0-IIc	0.0 (0.0)	0.0 (0.0)	-
Histopathology			
Adenoma with low-grade dysplasia	9.8 (25.0)	20.9 (35.7)	< 0.001
Adenoma with high-grade dysplasia	0.0 (0.0)	0.0 (0.0)	-
Invasive cancer	0.0 (0.0)	0.0 (0.0)	-
TSA	0.0 (0.0)	0.9 (9.7)	0.079
SSA/P	0.3 (5.5)	1.6 (11.7)	0.031
Hyperplastic polyp	0.0 (0.0)	0.0 (0.0)	-
ND	0.4 (3.8)	0.8 (8.4)	0.735

Data were presented as number of polyps per patient (SD).

*P*-value: Wilcoxon rank sum test.

ND: pathologic examination was not performed.

The adenoma miss rate per patient (AMR-PP) and ADR of adenoma diagnosed histopathologically were calculated. Histological analysis of PMR-PP revealed the AMR-PPs were 9.8% and 20.9% in the LCI-WLI and WLI-LCI groups, respectively (Table 4). Thus, similar to the PMR-PPs, the results significantly differed (Wilcoxon test, *P* < 0.001). In terms of the clinical features of the lesions (location, size, morphology, and histopathologic findings), similar results were obtained.

### Adenoma detection rate

The ADR in the first observation of the LCI-WLI group (ADR of LCI) and the ADR in the first observation of the WLI-LCI group (ADR of WLI) were 52.6% and 47.5%, respectively. Hence, the results did not significantly differ (odds ratio: 1.23 [95% confidence interval, CI: 0.90–1.67], *P* = 0.193) (Table 5). However, after stratifying for ADR based on lesion size, the ADR of LCI was significantly higher than that of WLI (38.2% vs. 29.1%, odds ratio: 1.51 [95% CI: 1.09–2.10], *P* = 0.014) in diminutive adenomas (< 5 mm). In terms of morphology, the ADR of LCI was significantly higher than that of WLI (39.8% vs. 31.9%, odds ratio: 1.41 [95% CI: 1.02–1.95], *P* = 0.038) in 0-Is lesions. Regarding location, there were no differences in ADRs between LCI and WLI.

**Table 5:** Adenoma detection rate (%).

	LCI-WLI group	WLI-LCI group	OR (95% CI)	*P*-value
	*n* = 327	*n* = 320		
All adenomas	172 (52.6)	152 (47.5)	1.23 (0.90, 1.67)	0.193
Location				
Ascending colon	83 (25.4)	68 (21.3)	1.26 (0.87, 1.82)	0.221
Transverse colon	66 (20.2)	55 (17.2)	1.21 (0.82, 1.81)	0.339
Descending colon	40 (12.2)	29 (9.1)	1.40 (0.85, 2.32)	0.191
Sigmoidal colon	78 (23.9)	73 (22.8)	1.06 (0.74, 1.53)	0.742
Rectum	28 (8.6)	25 (7.8)	1.11 (0.63, 1.95)	0.715
Size				
< 5 mm	125 (38.2)	93 (29.1)	1.51 (1.09, 2.10)	0.014
5–10 mm	95 (29.1)	85 (26.6)	1.13 (0.80, 1.60)	0.483
> 10 mm	39 (11.9)	42 (13.1)	0.90 (0.56, 1.43)	0.643
Morphology				
0-Is	130 (39.8)	102 (31.9)	1.41 (1.02, 1.95)	0.038
0-Isp	39 (11.9)	43 (13.4)	0.87 (0.55, 1.39)	0.077
0-Ip	12 (3.7)	17 (5.3)	0.68 (0.32, 1.44)	0.313
0-IIa	41 (12.5)	33 (10.3)	1.24 (0.76, 2.02)	0.384
0-IIb	0 (0.0)	0 (0.0)	-	-
0-IIc	0 (0.0)	0 (0.0)	-	-

The Bonferroni’s adjustment is applied for stratified analysis by factor.

Data were presented as number of participants (%).

OR: odds ratio.

*P*-value: Logistic regression analysis of covariates such as the endoscopists’ years of experience and number of endoscopy procedures performed.

All adverse events were caused by EMR (*n* = 1, perforation and *n* = 3, bleeding after EMR), not by the protocol design of this study.

## Discussion

In this study, LCI was found to reduce PMR in the whole colon comparing with WLI. However, the advantage of LCI was not demonstrated in the ascending and sigmoid colons. In addition, similar results on AMR-PP were obtained.

Min et al.^26^ evaluated the PMR of LCI and WLI via univariate and multivariate analyses. The results showed that the PMR of LCI was lower than that of WLI only in the descending colon (odds ratio: 4.444 [95% CI: 1.102–17.925], *P* = 0.0360, 5.415 [95% CI: 1.210–24.243], *P* = 0.0272). However, their sample size was too small to evaluate each segment of colon. Paggi et al.^27^ reported that LCI could reduce the AMR of neoplastic lesions in the right colon using the same design used (AMR: 11.8% vs. 30.6% in the LCI-WLI and WLI-LCI groups, respectively) in a single-center study. However, their result contradicts ours. This discrepancy might be attributed to the difference in the percentage of large lesions (> 10 mm) (15/566 vs. 132/1177). Previous studies revealed that IEE is effective in identifying diminutive lesions (< 5 mm)^14–16,18–20^. Furthermore, AMR and PMR decrease with greater lesion size in colonoscopy^4,5^. However, in our study, LCI reduced the PMR-PP and AMR-PP in relatively large adenomas (5–10 mm). Tanaka et al. compared ADR and PDR using LCI and WLI observations and similarly reported that the LCI observation group showed significantly higher rates for lesions 10 mm or smaller^35^. We believe that this is one of the advantages of LCI in the detection of colorectal lesions.Based on our results, we assumed that the efficacy of LCI is similar to that of WLI in the sigmoid and ascending colons. We believe that this is associated with the characteristics of each colon. Unlike any other colon segments, the sigmoid colon is indented and twisted frequently; therefore, there are several blind spots behind the folds. In the ascending colon, the deep gaps between the folds cause blind spots. Thus, LCI is advantageous as it has a bright view when assessing the lesions in long distance. However, it could not improve blind spots. To reduce the number of missed lesions above the blind spots, special colonoscopy such as wide-angle colonoscopy^9,10^, Third Eye colonoscopy^11,12^, and FUSE^13,14^ might be useful. However, none of the abovementioned colonoscopy procedures are equipped with LCI. By contrast, a transparent cap can be adapted in any type of colonoscopy procedure. Therefore, the combination of transparent cap and LCI should be evaluated for further studies.

In addition, the use of LCI is effective in 0 − Is and 0 − IIa lesions (*P* < 0.001, 0.024) (Table 4). Flat-type tumors are more likely to be detected in IEE than in WLI^19,27^. Our result was similar to previous reports.

PMR-PP was also analyzed by polyp histological type; however, lesions other than adenomas were found in insufficient numbers to permit statistical verification.

The withdrawal time in LCI was longer than that in WLI (6.66 vs. 6.38 min, *P* = 0.016). This is presumed to be due to the slightly higher number of polyps detected during LCI observation. The number of polyps detected during the first observation in the LCI-WLI group tended to be higher than that detected in the WLI-LCI group. Furthermore, cases with multiple polyps also tended to be more common in the LCI-WLI group (Supplementary Table 2). It is estimated that the time required for polyp detection is equivalent between LCI and WLI.

Additionally, we analyzed PMR-PP by skill level, however in this study, only 6 out of 45 participants were non-experts. In the experts, similar to the analysis of all endoscopists, the PMR-PP in the LCI-WLI group was significantly higher than that in the WLI-LCI group (3.6% vs. 7.6%, *P* = 0.438) (Supplementary Table 3). However, in non-experts, no significant difference in PMR-PP was observed between the LCI-WLI group and the WLI-LCI group (3.6% vs. 7.6%, *P* = 0.438) (Supplementary Table 4). In this study, the sample size was too small to draw significant conclusions regarding PMR-PP among non-experts, because only 25 cases were reviewed by non-experts across both groups.

Several studies adopted the PMR/AMR, which was defined as the number of total missed lesions in the first observation in all participants/the number of total detected lesions in all patients^3–5,9,18,20,26^. In the current study, we analyzed the PMR-PP, which was calculated as the number of newly detected polyps during the second observation/the number of total detected polyps during the first and second observations. There were three reasons in selecting PMR-PP. First, the polyps observed in the same patients could be correlated. Hence, the condition of data independence (statistical term; identically and independently distributed) for statistical tests was not satisfied. Second, randomized patients should be included in the analysis according to the intension to treat principle. Patients without polyps or missed polyps should not be excluded from the statistical analysis. Third, the patient, not the polyp, is the experimental unit in statistics. Thus, the patients were randomized, not the polyps. The selection of an analytical method presumed to be statistically more accurate is a strong point of this study.

ADR, which is the secondary endpoint, was assessed. Results did not show significant differences between LCI and WLI (52.6% vs. 47.5%, *P* = 0.193). ADR is the most reliable quality indicator in screening colonoscopy studies. However, recent studies with a high-resolution scope showed that ADRs greater than 50%^38^, and the differences were more likely to be less or none in studies comparing novel IEEs^18,19,21^. Min et al.^26^ reported that the ADR of LCI was higher than that of WLI (37% vs. 28%; [95% CI: 2.39%–19.41%]), even though each ADR was < 50%. The difference in ADRs in several studies might be attributed to the background characteristics of enrolled patients. Thus, we analyzed ADRs after stratifying based on adenoma size, location, and morphology. Thus, in diminutive adenoma, the ADR of LCI was significantly higher than that of WLI (39.4% vs. 30.0%, *P* = 0.012). The expectation for stratifying ADR based on various factors is presented in the review of the ASGE/ACG Task Force on Quality in Endoscopy^38^. In this study, we found the practicability of analysis stratified based on various characteristics of the lesions. In the future, the quality indicators for evaluating techniques and devices for the detection of colorectal polyps should be analyzed based on various factor with an adequate sample size.

The current study had several limitations. First, this study encompasses all colonoscopies performed in routine clinical practice and does not focus solely on surveillance colonoscopies. Therefore, this study is not a pure surveillance colonoscopy study. Sccond, some patients with polyps who were diagnosed at another institution were enrolled in this study. This might have caused the ADR to increase, and resulting in the absence of difference between LCI and WLI. Furthermore, information regarding the number of lesions detected in the previous examination may have introduced bias into the detectability of this study. However, since the primary outcome of this study is the miss rate, we believe the impact on the conclusion is minimal. Third, although polyps (< 10 mm) diagnosed macroscopically as hyperplastic polyp were excluded, we could not deny that adenoma was included among them completely. Although non-expert participation was limited in this study, distinguishing adenomas from hyperplastic polyps can be challenging in some cases. However, the number of related polyps was low in all segments of the colon except in the sigmoid colon. Hyperplastic polyps in the sigmoid colon and rectum tend to be multiple, making accurate counting difficult. Threfore, we determined that the PMR calculated including these polyps was not accurate. Furthermore, since hyperplastic polyps are generally not candidates for endoscopic resection, we believe the PMR calculated excluding them has clinical significance. Fourth, this study evaluated only the total score for BBPS. Therefore, cases with partially poor bowel preparation may also be included in the study. However, since we also used the Modified Aronchick Scale to evaluate the bowel preparation of the entire colon, we believe that cases with excessively poor cleansing were excluded. Fifth, inally, newer generations of colonoscopes than those used in this study are currently available, and compared to high-resolution colonoscopes, PMR and AMR may have been overestimated in this study. Finally, because only a small number of examinations were performed by non-experts in this study, the analyses within the non-expert group lacked sufficient statistical power.

This study demonstrated that LCI is effective in assessing small lesions in terms of PMR-PP, AMR-PP and ADR. However, a recent study employing newer endoscope have demonstrated no reduction in the rate of missed right-sided colonic lesions with LCI^39^. Moreover, a recent systematic review concluded that although LCI enhances ADR, it does not significantly influence lesion size, morphology, or location^40^. Given the heterogeneity in study design and outcome definitions, the overall efficacy of LCI remains difficult to establish. However, the clinical implementation of artificial intelligence (AI) is expected to unlock the full potential of IEE. Miyaguchi et al. have demonstrated that AI-assisted LCI observation improves both ADR and adenoma per colonoscopy (APC) compared with conventional LCI^41^. These findings highlight the need for further investigations incorporating AI in future studies.

In conclusion, LCI could improve PMR-PP in total colonoscopy, and this was significant in the transverse and descending colons and rectum. In addition, this technique could improve ADR in diminutive adenomas. Thus, it is more suitable for screening colonoscopy than WLI.

## Methods

### Patients and study design

This two-armed, randomized, back-to-back study was conducted from November 2018 to May 2020 by GI-Kyushu, a clinical research organization involving eight university hospitals and eight academic centers in the Kyushu region of Japan. The study protocol was registered on the Japan Registry of Clinical Trials (jRCTs072180006) (12/11/2018). The present study was conducted by the Declaration of Helsinki of 1964 and its subsequent amendments or equivalent ethical standards.

The central institution has a licensing committee/institutional review board (IRB) to approve human subjects research. The research protocol obtained approval from the NPO Clinical Research Network Fukuoka Certified Review Board (11/10/2018). The central institution’s IRB review was applied to this study, and approval was obtained from all participating hospitals’ ethics committees and IRB (Supplementary).

All research was performed according to relevant guidelines/regulations, and informed consent was obtained from all participants.

Patients aged 18–79 years who were referred and scheduled for total colonoscopy in 16 institutions were prospectively enrolled in this research. Prior to the procedure, all patients provided informed consent. The inclusion criteria were as follows: patients with positive fecal immunochemical test (FIT) results and those with a previous history of colorectal neoplasms, polyp surveillance, and any abdominal symptoms. Meanwhile, patients with inflammatory bowel disease; familial history of polyposis syndrome (e.g., familial adenomatous polyposis, juvenile polyposis, and hereditary non-polyposis CRC [Lynch syndrome]) and history of surgical resection of any part of the colon; and poor general condition and those who cannot provide informed consent were excluded. Patients received lavage with 2–3 L of polyethylene glycol or 1.8 L of magnesium citrate in the morning on the day before colonoscopy until rectal fluid was clear and cleansing enema. After enrollment, the participants were excluded if they could not be intubated to the cecum during colonoscopy and/or if bowel preparation was poor/inadequate based on the Modified Aronchick Scale or Boston Bowel Preparation Scale (BBPS) (score of < 5).

### Endoscopic equipment and setting

The LASEREO system (light source: LL4450/7000, processor: VP4450HD/7000, Fujifilm Co., Japan) and a high-definition monitor were used. All procedures were performed using a high-resolution video colonoscope (EC-L600ZP/L600ZP7/L600MP7 Fujifilm Co., Japan).

### Endoscopic procedures

The same endoscopist performed same-day, back-to-back tandem colonoscopy. They were randomly assigned to either the LCI-WLI or WLI-LCI group during withdrawal. In the LCI-WLI group, the colonoscope was removed from the cecum to the hepatic flexure in LCI (first observation) and was reinserted into the cecum. Thereafter, the colonoscope was withdrawn again in WLI (second observation). Each colon segment (ascending, transverse, descending, and sigmoid colons and rectum) was examined twice, once with LCI and once with WLI. In the WLI-LCI group, the same steps were performed with changes in the order of LCI and WLI. During the entire withdrawal phase, each colon segment was comprehensively assessed for the presence of lesions. In total, 45 endoscopists (39 expers and 6 non-experts) participated in this study. Endoscopists with experience of more than 1000 colonoscopic procedures were defined as exparts, while those with fewer experience were defined as non-experts. The colonoscopists’ median (min–max) years of experience was 9.0 (0.5–26.0), and the mean number of colonoscopic procedures performed was 2100 (200–13000). All had previous experience with high-resolution colonoscopy and that equipped with LCI. Before examination, the endoscopists were blinded to information regarding colorectal polyps.

The target lesions include premalignant lesions (adenoma, dysplasia, and serrated lesions) and carcinoma based on assessment using the World Health Organization guidelines^42^. In serrated lesions, sessile serrated adenoma/polyp (SSA/P) and traditional serrated adenoma (TSA) were considered as target lesions. However, serrated lesions (< 10 mm) considered macroscopically as hyperplastic polyp were excluded because they increase in number. The characteristics of all lesions including location, size, and morphology were documented during each examination. The lesion size was compared with that of open endoscopic biopsy forceps. To classify the macroscopic morphology of the detected polyps, we used the Paris classification of superficial gastrointestinal lesions^43^. All lesions except for the hyperplastic polyps diagnosed endoscopically in the sigmoid colon and rectum underwent endoscopic resection followed by histopathological examination.Moreover, they were pathologically assessed according to the Japanese classification for colorectal carcinoma^44^. In this study, neoplasms were classified into three categories, which were as follows: adenoma with low-grade dysplasia, adenoma with high-grade dysplasia, and invasive cancer. These categories corresponded to categories 3, 4 and 5 in the Vienna classification^45^, respectively.

### Outcome measure

The primary endpoint was the difference in PMR-PPs between the LCI-WLI and WLI-LCI. In the current study, we analyzed the PMR-PP, which was calculated as the number of newly detected polyps during the second observation/the number of total detected polyps during the first and second observations.

The secondary endpoint was ADR, and the adenoma miss rate per patient (AMR-PP) was used as the endpoint of the sensitivity analysis of PMR-PP. AMR-PP and ADR were calculated if the lesions were diagnosed as adenoma based on pathological findings.

### Sample size calculation

The sample size was calculated using the chi-square test to assess the proportion of participants with at least one missed adenoma for simplicity. However, the primary endpoint was analyzed using the Wilcoxon rank sum test. At the time of the study design planning, no reports existed regarding the miss rate of LCI for colorectal polyps. Therefore, the sample size was calculated by referencing a study that used BLI, another IEE employing the same laser light source, to calculate the miss rate using a similar study design. Shimoda^18^ reported that the probabilities in which the missed adenoma is detected were 10% in the WLI-WLI group and 1.6% in the BLI-WLI group. Based on this result, the detection probability per adenoma in WLI and BLI were about 88.7% and 98.2%, respectively. Assuming that WLI and BLI are similar in terms of detecting adenomas, the probabilities in which the missed adenoma is detected were 11.1% in the WLI-LCI group and 1.6% in the LCI-WLI group. Some patients present with multiple adenomas, and the probabilities in patients with at least one missed adenoma were conservatively set at 13% and 5%, respectively. A group of 200 was required to achieve a two-sided significance level of 5% and power of 80%. Hence, 400 patients with adenomas were recruited.

### Randomization and monitoring

Permuted block randomization, stratified according to institution, with a block of size six was performed using an electric data capture system (EDC: Viedoc™, version 4.58, Viedoc Technologies) with 1:1 allocation. Until randomization, the endoscopists were blinded to the allocation group. Central monitoring was performed once to evaluate for safety, study discontinuation and progression, and protocol deviations after 400 participants were enrolled. To confirm if this study was performed according to protocol and if data were collected timely and accurately, central monitoring was performed by the data manager, principal investigator, and research officer.

### Statistical analysis

All variables were presented as medians and ranges (min–max), means, and standard deviations or numbers (%). The PMR-PP and AMR-PP were compared between groups using the Wilcoxon rank sum test. ADR was calculated as 100*number of participants with adenoma detected in the first pass/number of participants. Then, logistic regression analysis was performed using covariates such as years of experience in colonoscopy and number of colonoscopy procedures performed.

The PMR-PP, AMR-PP, and ADR were also analyzed based on lesion location, size, and morphology with the same analysis method used for each endpoint. A *P*-value of < 0.05 was considered statistically significant. The Statistical Analysis System version 9.4 (SAS Institute, Cary, NC, the USA) was used for all analyses.

## Supplementary Information

Below is the link to the electronic supplementary material.

Supplementary Material 1

## Data Availability

The datasets generated and/or analyzed during the current study are not publicly available due to privacy and ethical restrictions but can be requested and reviewed from the corresponding author on reasonable request.
