# Supplementary material for: Linked color imaging improves polyp miss rates in total colonoscopy in a multicenter randomized back to back trial
Source: Sci Rep. 2025 Oct 21;15:36788. doi: 10.1038/s41598-025-20633-2 (PMC12541007; doi:10.1038/s41598-025-20633-2)
Supplement: Supplementary file 1 — Supplementary Material 1 [file 41598_2025_20633_MOESM1_ESM.docx]

**Supplementary Tables**

Table S1. Modified Aronchick Scale (MAS)

| Grade | Description |
| --- | --- |
| Excellent | Small amount of clear liquid with clear mucosa seen; more than 95% mucosa seen |
| Good | Small amount of turbid fluid without faeces, not interfering with examination; more than 90% mucosa seen |
| Fair | Moderate amount of stool that can be cleared with suctioning to permit adequate evaluation of entire colonic mucosa; more than 90% mucosa seen |
| Poor | Inadequate but examination completed; enough faeces or turbid fluid to prevent a reliable examination; less than 90% mucosa seen |
| Inadequate | Re-preparation required; large amount of faecal residue precludes a complete examination |

This scale evaluates the entire intestinal tract reported in descending order of cleansing degree on a 5-point scale: excellent, good, fair, poor, and inadequate.

Table S2. Number of polyps detected at the first observation

|  | LCI-WLI group | WLI-LCI group | *P*-value |
| --- | --- | --- | --- |
|  | n = 327 | n = 320 |  |
| 0 | 138 (42.2) | 157 (49.1) | 0.025 |
| 1 | 69 (21.1) | 67 (20.9) |  |
| 2 | 43 (13.1) | 48 (15.0) |  |
| 3-5 | 56 (17.1) | 37 (11.6) |  |
| 6- | 21 (6.4) | 11 (3.4) |  |

Data represent the number of patients (%)

P-value: Wilcoxon rank sum test.

Table S3. Polyp miss rates (%) per patient (SD) in experts

|  | LCI-WLI group | WLI-LCI group | *P*-value |
| --- | --- | --- | --- |
|  | n = 313 | n = 309 |  |
| All polyps | 9.6 (23.9) | 21.0 (34.4) | <0.001 |
| Location |  |  |  |
| Ascending colon | 4.5 (18.7) | 8.6 (26.9) | 0.048 |
| Transverse colon | 4.0 (18.1) | 11.5 (29.9) | <0.001 |
| Descending colon | 2.8 (15.5) | 7.6 (25.7) | 0.009 |
| Sigmoidal colon | 4.9 (19.8) | 6.5 (23.2) | 0.585 |
| Rectum | 1.0 (9.8) | 3.2 (17.3) | 0.029 |
| Size |  |  |  |
| <5 mm | 9.7 (26.3) | 17.3 (34.2) | 0.003 |
| 5–10 mm | 4.1 (17.3) | 11.0 (28.5) | 0.002 |
| >10 mm | 0.2 (3.8) | 1.1 (10.0) | 0.098 |
| Morphology |  |  |  |
| 0-Is | 7.6 (21.0) | 17.8 (34.0) | <0.001 |
| 0-Isp | 0.6 (8.0) | 2.1 (12.9) | 0.032 |
| 0-Ip | 0.0 (0.0) | 0.3 (5.7) | 0.314 |
| 0-IIa | 2.6 (14.1) | 6.2 (22.5) | 0.026 |
| 0-IIb | 0.0 (0.0) | 0.0 (0.0) | - |
| 0-IIc | 0.0 (0.0) | 0.0 (0.0) | - |
| Histopathology |  |  |  |
| Adenoma with low-grade dysplasia | 10.0 (25.4) | 21.4 (36.2) | <0.001 |
| Adenoma with high-grade dysplasia | 0.0 (0.0) | 0.0 (0.0) | - |
| Invasive cancer | 0.0 (0.0) | 0.0 (0.0) | - |
| TSA | 0.0 (0.0) | 1.0 (9.8) | 0.081 |
| SSA/P | 0.3 (5.7) | 1.7 (11.9) | 0.032 |
| Hyperplastic polyp | 0.0 (0.0) | 0.0 (0.0) | - |
| ND | 0.4 (3.9) | 0.8 (8.3) | 0.727 |

Data were presented as number of polyps per patient (SD).

*P*-value: Wilcoxon rank sum test.

ND: pathologic examination was not performed.

Table S4. Polyp miss rates (%) per patient (SD) in non-experts

|  | LCI-WLI group | WLI-LCI group | *P*-value |
| --- | --- | --- | --- |
|  | n = 14 | n = 11 |  |
| All polyps | 3.6 (13.4) | 7.6 (17.3) | 0.438 |
| Location |  |  |  |
| Ascending colon | 7.1 (26.7) | 0.0 (0.0) | 0.375 |
| Transverse colon | 0.0 (0.0) | 0.0 (0.0) |  |
| Descending colon | 0.0 (0.0) | 0.0 (0.0) |  |
| Sigmoidal colon | 0.0 (0.0) | 12.1 (30.8) | 0.103 |
| Rectum | 0.0 (0.0) | 0.0 (0.0) |  |
| Size |  |  |  |
| <5 mm | 0.0 (0.0) | 9.1 (20.2) | 0.103 |
| 5–10 mm | 7.1 (26.7) | 0.0 (0.0) | 0.375 |
| >10 mm | 0.0 (0.0) | 0.0 (0.0) |  |
| Morphology |  |  |  |
| 0-Is | 0.0 (0.0) | 9.1 (20.2) | 0.103 |
| 0-Isp | 7.1 (26.7) | 0.0 (0.0) | 0.375 |
| 0-Ip | 0.0 (0.0) | 0.0 (0.0) |  |
| 0-IIa | 0.0 (0.0) | 0.0 (0.0) |  |
| 0-IIb | 0.0 (0.0) | 0.0 (0.0) |  |
| 0-IIc | 0.0 (0.0) | 0.0 (0.0) |  |
| Histopathology |  |  |  |
| Adenoma with low-grade dysplasia | 3.6 (13.4) | 7.6 (17.3) | 0.438 |
| Adenoma with high-grade dysplasia | 0.0 (0.0) | 0.0 (0.0) |  |
| Invasive cancer | 0.0 (0.0) | 0.0 (0.0) |  |
| TSA | 0.0 (0.0) | 0.0 (0.0) |  |
| SSA/P | 0.0 (0.0) | 0.0 (0.0) |  |
| Hyperplastic polyp | 0.0 (0.0) | 0.0 (0.0) |  |
| ND | 0.0 (0.0) | 0.0 (0.0) |  |

Data were presented as number of polyps per patient (SD).

*P*-value: Wilcoxon rank sum test.

ND: pathologic examination was not performed.
